# A Rare Case of Penile Strangulation Managed by Orthopedic Jumbo Cutter

**DOI:** 10.7759/cureus.15659

**Published:** 2021-06-15

**Authors:** Jayanta K Laik, Abhay H Kerketta, Ritesh Kumar, Ravi Kausal, Ajay Agarwal, Manoj Rajak

**Affiliations:** 1 Department of Joint Replacement and Orthopedics, Tata Main Hospital, Jamshedpur, IND; 2 Department of Urology, Tata Main Hospital, Jamshedpur, IND

**Keywords:** penile strangulation, metallic ring, jumbo cutter, orthopedic surgeon, urology

## Abstract

Penile strangulation is not commonly encountered in orthopedic practice. Quick decision and immediate removal of the metallic ring with readily available instruments is the key to a successful outcome. Jumbo cutter is a commonly available instrument. It can be used with ease without causing any thermal or soft tissue damage, giving satisfactory results.

## Introduction

Penile strangulation is a rare entity in urological emergencies and not commonly encountered in orthopedic practice. Usually, orthopedic surgeons come to the picture when urologists fail to remove and ask help from orthopedists as they deal with hardware removal. Quick decision and immediate removal of the metallic ring with a readily available instrument is the key to a successful outcome. Jumbo cutter is a commonly available instrument in the orthopedics armamentarium. It can be used with ease without causing any thermal or soft tissue damage, giving satisfactory results. Here, we report a case of penile strangulation caused by a metallic ring which was treated successfully with an orthopedic jumbo cutter.

Strangulation of the penis is one of the rare and challenging urological emergencies [1]. This type of strangulation results from various metallic and non-metallic objects [2]. Non-metallic objects can be easily removed, however, metallic objects like rings are very difficult to remove without causing penile damage [3]. Good results are often obtained when a patient presents early but if delayed, there is edema and venous congestion to the distal part. The removal of the foreign body becomes difficult due to edema and often causes damage to the underlying tissue [4].

Most often, this emergency is dealt with by either a urological or surgical team and very rarely orthopedic surgeon comes in for the rescue. There are various methods described in literature like the string method and the use of various cutting devices. Cutting devices described are an iron saw, diamond-tipped dental drill, and other orthopedic tools [5,6]. The powered gadget causes thermal damage due to excessive heat generation and is not available readily in emergencies. Handling of these powered gadgets needs skilled expertise too [7,8].

## Case presentation

**Figure 1:**
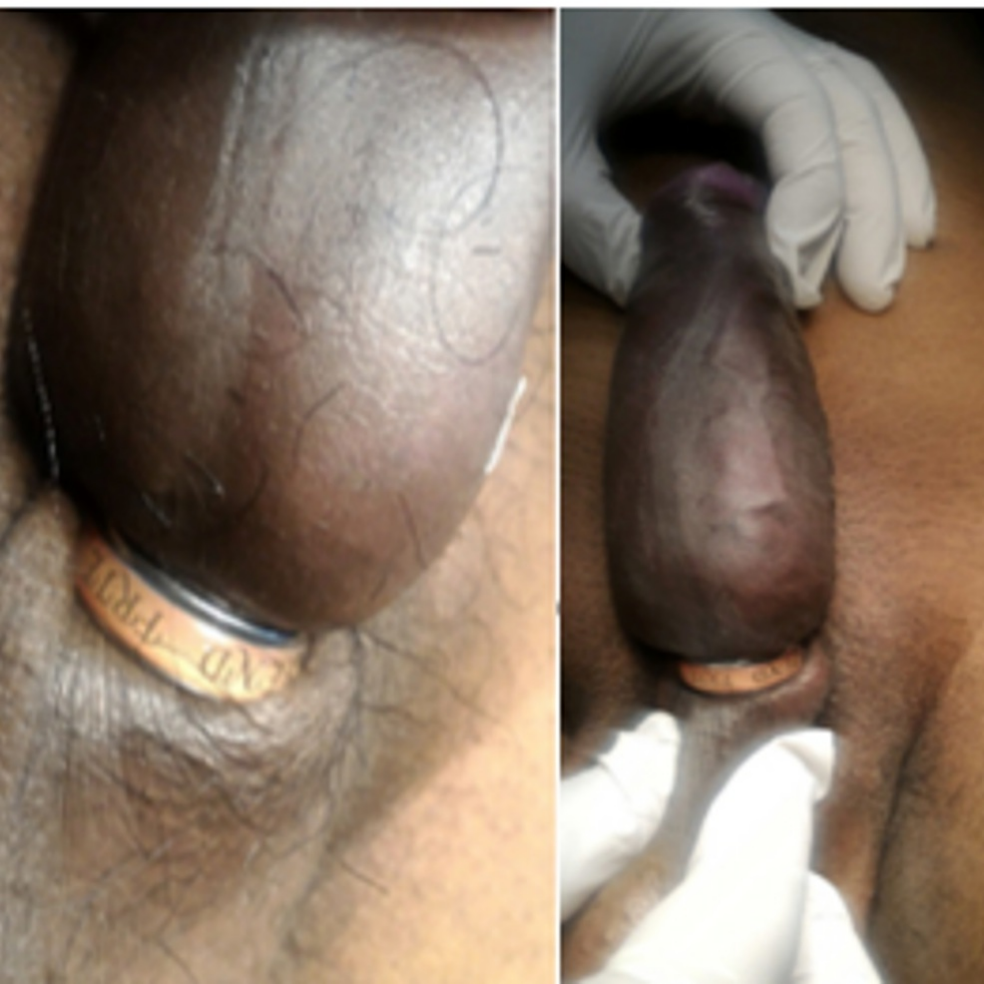
Emergency presentation of metal ring stuck at the base of the penis with significant distal edema.

A 25-year-old male presented to emergency with a history of metallic ring stuck at the base of penis following insertion for sexual pleasure, two hours after the incident (Figure 1). Initially, he was admitted to the urology department and was posted for its removal. Help was sought when they failed to remove it after their best efforts. The ring was approximately 1 cm wide and 0.25 cm thick and was made up of gold-plated platinum. The penis was swollen with a constriction at the base. The venous prominence was seen distal to the constriction ring. Mild edema at the glans penis was noted. The first attempt was made to cut the ring using a motorized cutting tool used in orthopedics. However, the speed and size of the saw were worrisome and there was every possibility of damaging the underlying soft tissue due to its high speed. Hence, this attempt was abandoned.

**Figure 2:**
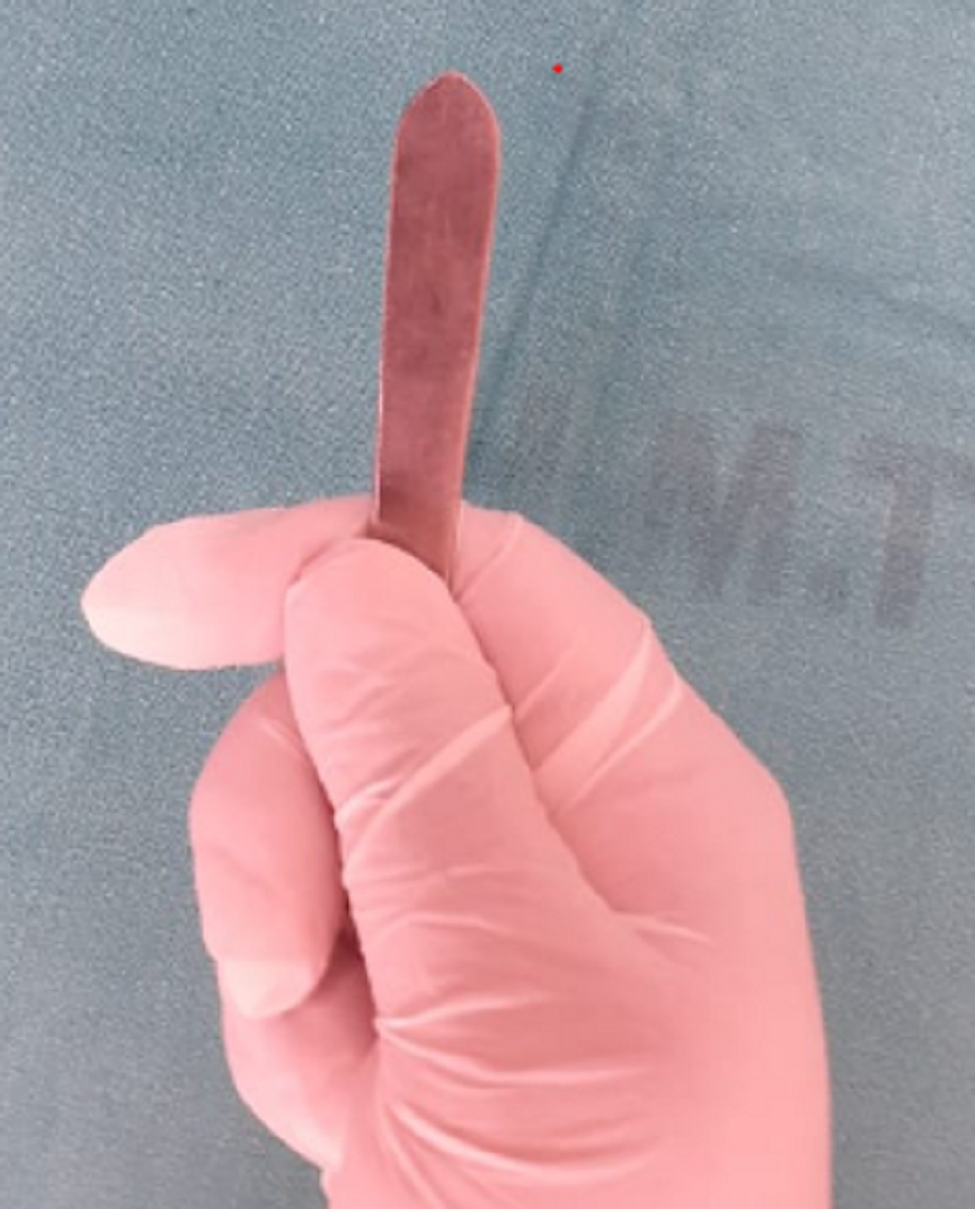
Reverse end of Bard Parker handle which was insinuated between ring and penis.

**Figure 3:**
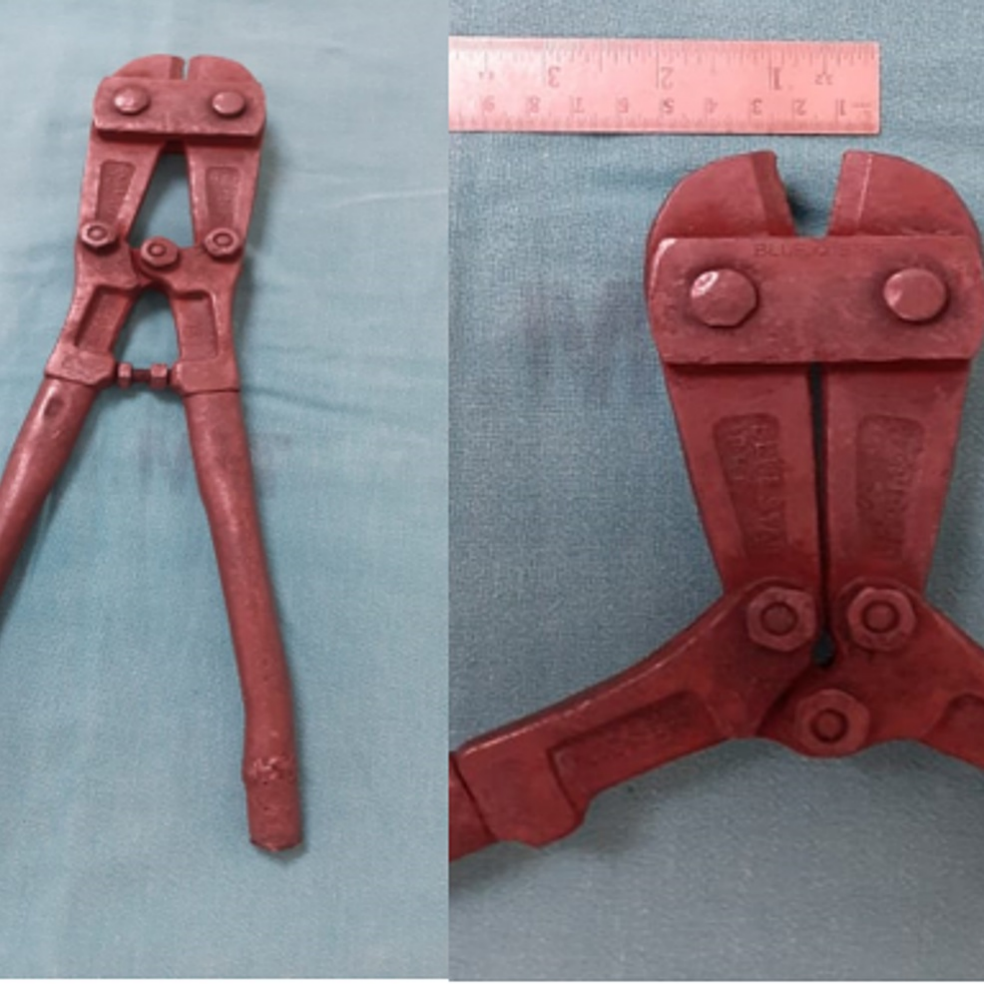
Picture on the left - jumbo cutter used to cut the ring. Picture on the right - the jaw of the cutter can open out to 1.5 cm as shown in the image.

**Figure 4:**
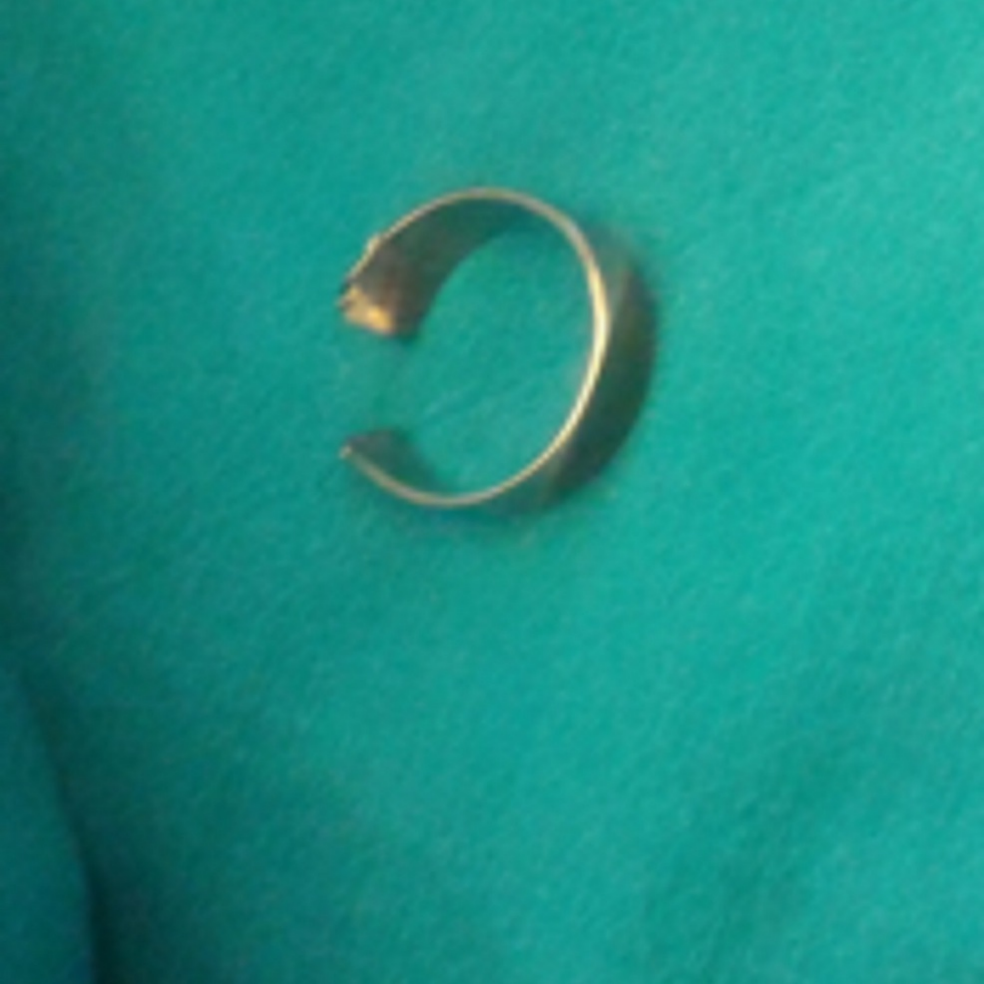
Cut ring retrieved after prying the ends open with nose plier.

**Figure 5:**
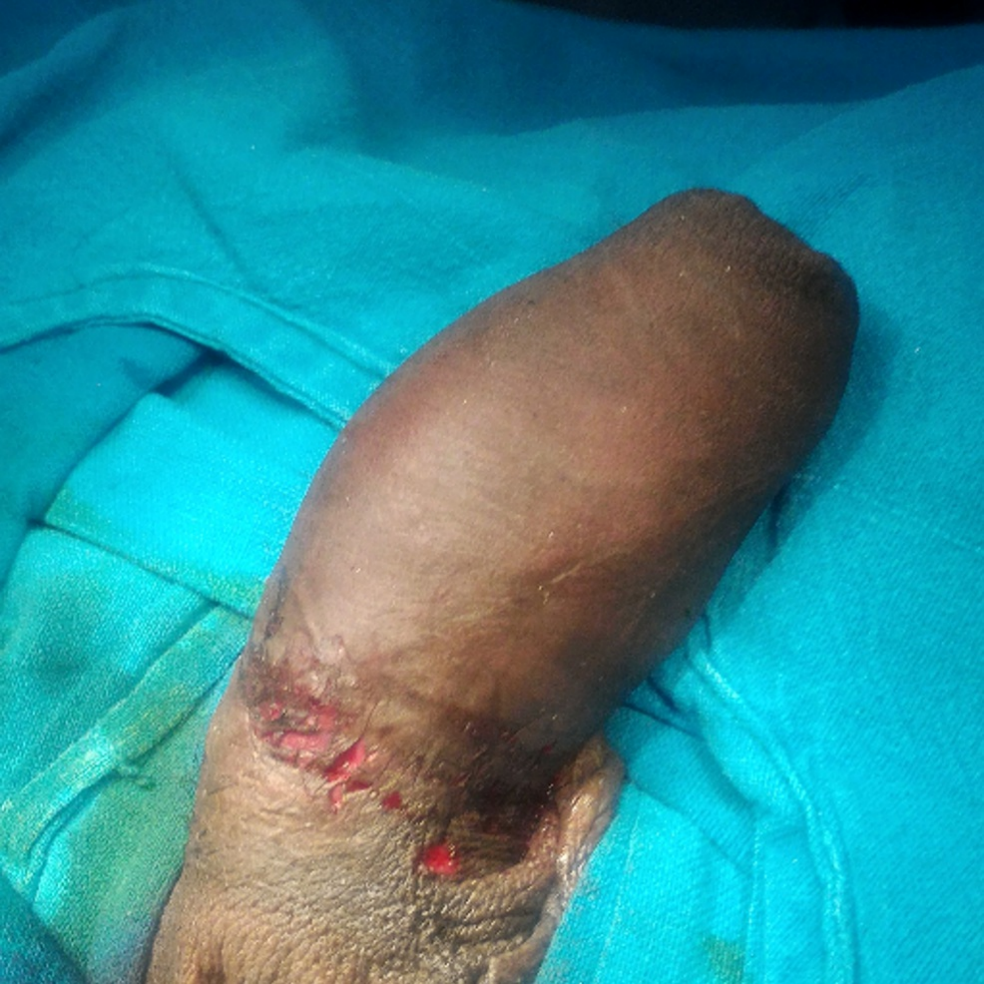
The penis immediately after removal of the ring showing superficial skin abrasion over its base.

The second attempt was made using a Bard-Parker (BP) handle (Figure 2) and a jumbo cutter (Figure 3). This technique was fairly simple, as we passed the reverse end of the BP handle between the metallic ring and soft tissue at the base of the penis. The jumbo cutter was used to cut the ring over it. The advantage of using the reverse end of the BP handle blade was to ensure the prevention of soft tissue damage as one stabilizes it, while the other person can use a considerable amount of force over the jumbo cutter. After cutting the ring, the ends were opened out (Figure 4) using nose pliers and the penis was freed with minimal soft tissue damage (Figure 5) within an hour of emergency posting. The venous engorgement was quickly subsided and edema subsided after an hour.

**Figure 6:**
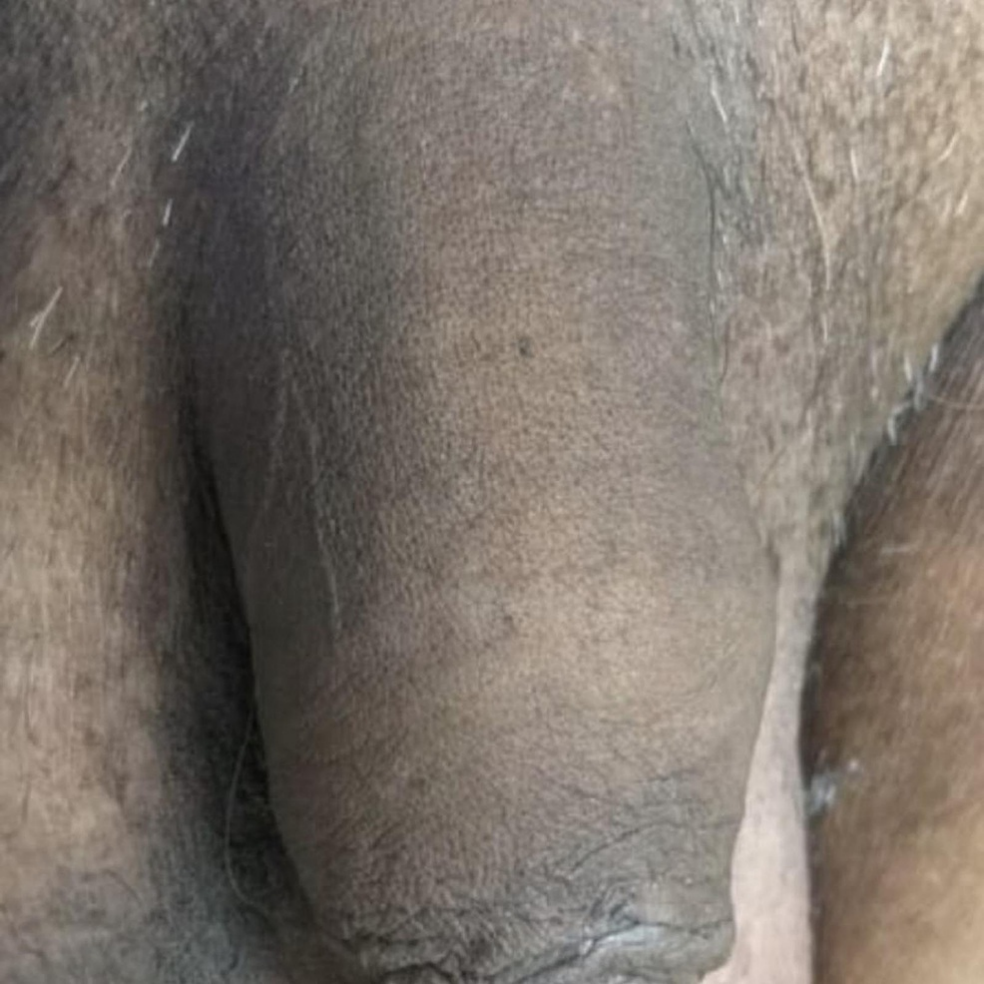
At one-year follow-up, the penis had no residual sequelae.

The case was followed up in urology OPD at regular intervals. At follow-up one year later, the penis showed complete healing of ventral skin abrasions and no residual constriction (Figure 6).

## Discussion

It is very unusual to see a foreign body like the metallic ring to be inserted at the base of the penis. These acts are seen often in children where the reason often is accidental or curiosity. In adults, though, seen in the enhancement of erection, prolongation of the sexual activity as well as in mentally unstable adult males as mentioned by Marzorati and Ascione [1]. The penis is a sensitive and delicate organ and the entrapment of the ring can cause venous obstruction in an erected penis. Venous congestion leads to further edema. This may result in circulatory impedance and gangrene of the distal part of the penis. Early removal of the ring breaks the vicious cycle and can restore normal function [9]. Late treatment may cause varying degrees of incarceration which may result in skin ulceration and damage to corpus spongiosa with or without urethral damage. In severe cases, there might be loss of distal penile sensation, complete division of corpus spongiosa which might lead to urethral fistula, gangrene, or auto amputation of the distal penis [10].

Removal of the metallic ring is very challenging and there are multiple ways to extract the foreign body mentioned in the literature [11]. Below are the various methods of extraction described by different authors with varied results (Table 1) [11-35].

**Table 1:** Table showing reviewed literature with methods of extraction and outcome

Author and Journal	Department	Methods of extraction	Result
Marzorati and Ascione [1]	Department of Urology	Not mentioned	Urethro-cutaneous fistula
Gupta et al. [12]	Department of Surgery	Tourniquet compression	Good
Efthimiou et al. [13]	General Hospital of Chania, Greece	Angle grinder	satisfactory
Baruah et al. [2]	Department of Urology	Circumcoronal incision	satisfactory
Darby et al. [14]	Department of Urology	Midas-Rex pneumatic drill with metal cutting carbide attachment	satisfactory
Farooqui and Meena [5]	Department of Medicine, Surgery	Tourniquet compression	Good
Li et al. [15]	Department of Urology	Motor-operated emery wheel machine	satisfactory
Alkadri et al. [16]	Department of Urology	Cutting tool not mentioned	Not mentioned
Li et al. [15]	Department of Urology	Motor operated emery wheel machine	satisfactory
Alkhureeb [17]	Department of Urology	Lateral corpectomy with compression	satisfactory
Chih et al. [18]	Department of Emergency Medicine, Urology and Internal Medicine	Wire cutter	Satisfactory
Saha et al. [19]	Dept of Urology	Corporal aspiration followed by incision	Satisfactory
Choudhary et al. [20]	Department of Accident Emergency Medicine	Decompression with needle	Difficulty in erection and fibrosis of shaft of penis
Kouka et al. [21]	Department of Urology	Probe Foley CH 16	Gangrene
Shukla et al. [22]	Department of Surgery	Various techniques	Three satisfactory, two developed skin necrosis, one minor skin injury, one absconded
Goyal et al. [23]	Department of Surgery	Penile aspiration and string technique	Satisfactory
Albahri et al. [24]	Department of Maxillofacial Surgery, Department of Paediatric Surgery	Dental burrs diamond cutting disc	Satisfactory
Purnell et al. [25]	Department of Urology	Midas Rex Legend pneumatic orthopedic drill with metal cutter	Satisfactory
Fhima and Lahouel [8]	Department of Casualty, Department of Plastic, Reconstructive and Aesthetic Surgery	Aspiration Method with multiple punctures	Satisfactory
Tavukçu et al. [26]	Department of Urology	Manual decompression with multiple punctures	Satisfactory
Paonam et al. [9]	Department of Urology, Dentistry, Surgery	Micromotor	Good
Kumar et al. [10]	Department of Urology	K-wire cutter	Good
Matsumiya et al. [27]	Department of Urology, Dentistry	Airtime cutter	Not mentioned
Tavukçu et al. [26]	Department of Urology	Manual decompression with multiple punctures	satisfactory
Kanakarajagupta [28]	Department of General Surgery	Bone cutter	Satisfactory
Ichaoui et al. [29]	Department of Surgery	Angle grinder	Wet gangrene
Patel et al. [30]	Department of Urology	Industrial bolt cutter	good
Singh et al. [31]	Department of Urology and Department of Surgery	Motorised electric cutter	Penile dysfunction
Raja et al. [32]	Department of General Surgery	K-wire cutter	Satisfactory
Khan et al. [33]	Department of Urology	Plumber’s hacksaw	Satisfactory
Meena et al. [34]	Department of General Surgery	Electric saw	Satisfactory
Kumar et al. [4]	Department of Urology	Bolt cutter	Partial amputation of the penis
Vyas et al. [35]	Department of Surgery	Silk thread	Good
Sandeep et al. [11]	Department of Urology	Several methods	Mixed
Yu et al. [7]	Department of Surgery	Diamond disc cutter	Normal
Monib and Amr [7]	Department of General Surgery	Metal cutter	Satisfactory
Nason et al. [3]	Department of Urology, Emergency Medicine	Electric hand-operated axel grinder	Satisfactory

We reviewed literature from 2004 to 2019 which showed successful removal of the object with different methods. It was noted that 13 of the publications had satisfactory results using manual decompression with multiple punctures, metal cutter, or k wire cutter. However, the thickness of the ring and timing of removal of the ring was not available from the above publications. Various studies using other methods like micro motor, industrial bolt cutter, or k wire cutter have given good results. Air time cutters were also used with favorable results but their final outcome has not been mentioned. Angle grinders were also used to remove the ring, however, this patient developed wet gangrene later. Good results were also shown using silk thread for the removal of the ring. In some cases, motorized cutter, as well as open incision and removal of the ring, is also described. 

Our technique using BP handle and Jumbo cutter is a simple and innovative method. Jumbo cutter is readily available in the orthopedic operation theatre and can cut metal rings with relative ease without causing damage to underlying soft tissue. It is one of the standard tools to be kept in an emergency for the removal of metallic objects like rings, bangles, etc., more commonly from extremities and fingers. The use of this tool does not need much expertise. Thus, the golden hours are not lost causing irreversible damage to the internal structure of the genitalia.

We recommend the Jumbo cutter where the ring width is up to 1 cm, as it can accommodate well to get a good grip (Figure 3).

## Conclusions

It is a challenge for the orthopedic team to deal with such type of rare cases in practice. Various combinations and modifications of techniques have been described in the literature. Our technique for the removal of rings with a BP handle and Jumbo cutter stands out as it is very simple, effective, and readily available. It does not cause much underlying tissue damage either. We also recommend early reporting and a multi-disciplinary approach to deal with such cases. This case report is also aimed at creating awareness among surgeons to handle the instruments in a simple and easy way.
